# MicroRNA-Mediated In Vitro and In Vivo Direct Conversion of Astrocytes to Neuroblasts

**DOI:** 10.1371/journal.pone.0127878

**Published:** 2015-06-01

**Authors:** Maryam Ghasemi-Kasman, Maryam Hajikaram, Hossein Baharvand, Mohammad Javan

**Affiliations:** 1 Department of Physiology, Faculty of Medical Sciences, Tarbiat Modares University, Tehran, Iran; 2 Department of Stem Cells and Developmental Biology at Cell Science Research Center, Royan Institute for Stem Cell Biology and Technology, ACECR, Tehran, Iran; 3 Department of Developmental Biology, University of Science and Culture, ACECR, Tehran, Iran; Instituto Butantan, BRAZIL

## Abstract

**Background:**

The conversion of astrocytes to neuroblasts holds great promise for treatment of neurodegenerative and traumatic brain diseases.

**Methodology and Principal Findings:**

Here we have shown that adult human astrocytes could be reprogrammed to neuroblasts by miR-302/367, both in vivo and in vitro. However, the reprogramming of adult mouse astrocytes to neuroblasts required valproic acid (VPA), a histone deacetylase inhibitor. Following induction of astrocytes toward neurons the expression of pluripotency markers were not detected, which suggested direct cell conversion. We did not observed tumor formation during two months follow up.

**Conclusions and Significance:**

These results show that neuroblasts can be generated directly from adult human and mouse astrocytes by miR-302/367-driven induction. This approach seems promising for converting glial scar cells into neuroblasts in a wide range of neurological diseases.

## Introduction

Following brain injury, multipotent neural stem cells (NSCs) and neural progenitors proliferate, migrate and produce new neural cells that contribute to the repair of lesions. There are two main pools of NSCs in the adult brain—NSCs in the subventricular zone (SVZ) of the lateral ventricles and those in the subgranular zone (SGZ) of the hippocampus [1]. Although these cells play a pivotal role in repairing limited damages, their potential for repairing extensive lesions is limited [2]. Attempts are ongoing to overcome this bottleneck in order to enhance the endogenous repair potential of the central nervous system (CNS).

The emergence of induced pluripotent stem cells (iPSCs) by Yamanka and colleagues have revealed that the cellular program of differentiation is reversible when appropriate transcription factors are introduced into cells [3]. This finding introduced a new approach for producing all cell lineages form a small skin biopsy for autologous transplantation. To avoid passing the cells through the pluripotency stage and the subsequent iPSCs differentiation toward the desired cells, researchers have introduced direct reprogramming of somatic cells. The later strategy is expected to reduce the risk of teratoma formation reported following in vivo application of iPSCs [4]. Via direct reprogramming (transdifferentiation) a particular cell type can be directly reprogrammed to another cell without passing through the transitional pluripotent intermediate stage [5]. Numerous studies have demonstrated that different somatic cells can be directly reprogrammed to neural precursors and neurons [6–13].

As starting cells, astrocytes have been directly converted into neuron and neuroblasts using different transcription factors, both in vitro and in vivo [14–18]. Astrocytes are known as the main component of glial scars that occurs in neurodegenerative diseases and traumatic brain injuries. Despite the initial benefits, glial scar prevents axonal regeneration. In vivo transdifferentiation of astrocytes into neural precursors may be considered an option for effective regeneration and repair.

Small molecules and microRNAs—short single strand RNAs with an important role in gene expression regulation at post-transcriptional levels—have been widely used to enhance the efficiency of reprogramming via transcription vectors [19–24]. The miR-302/367 cluster has been frequently reported as one of the highly expressed microRNAs in pluripotent stem cells [25–29]. Morrisey et al. showed that the miR-302/367 cluster alone was enough to reprogram human fibroblasts to iPSCs, while the presence of valproic acid (VPA) as a histone deacetylase inhibitor was necessary for conversion of mouse fibroblasts to iPSCs by the miR-302/367 cluster. This observation was suggested to be the result of high level activity of histone deacetylases in mice [25]. Reprogramming via miR-302/367 might involve different pathways such as targeting several epigenetic factors that result in global demethylation of the genome, inhibition of Oct4 suppressor factors, and enhancement of pluripotency markers [28, 30–33].

Here, we have shown high conversion of astrocytes to neuroblasts by miR-302/367 administered in conjunction with VPA. Human astrocytes transduced with miR-302/367 produced neuroblasts in vitro as well as in vivo when engrafted into the adult mouse brain. These induced neuroblasts could potentially generate neuronal cells; thus miR-302/367 might be considered a new tool for conversion of glial scar astrocytes to endogenous neuroblasts in repairing lesions for different neurological diseases.

## Materials and Methods

### Animals

C57BL/6 male mice (Pasteur Institute, Tehran, Iran), 8–9 weeks of age (20–25 g) were kept in a temperature-controlled animal house under a 12-hour light/dark period. Animals had ad libitum access to food and water. All experiments were performed in accordance with the international guide for the care and use of laboratory animals. The experimental procedures were evaluated and approved by the Committee for Ethics in Animal Research at Tarbiat Modares University. Attempts were made to minimize the animals' suffering and the number of animals used.

### Viral particle preparation

We prepared the miR-302/367 cluster as lentiviral particles which included a GFP expressing sequence (System Biosciences, San Francisco, CA) by transfecting along with a Virapower Lentiviral Packaging Mix (Invitrogen) into 293T cells by the Lipofectamine 2000 Transfection Reagent (Invitrogen). At 48 hours after transfection**,** supernatants were collected, filtered**,** concentrated and re-suspended. Some of the viral particles were used to determine functional transfection potential by transfection of HEK cells in vitro.

### Human astrocyte culture and transfection

Human astrocytes (line 1321N1) [34] were seeded onto poly-L-lysine (Sigma-Aldrich) coated coverslips and cultured in Dulbecco's Modified Eagle's medium (DMEM, Invitrogen), 10% fetal calf serum (FCS,Invitrogen) and 1X penicillin/streptomycin (Invitrogen). The culture medium was changed every three days. The purity of astrocyte cells was checked by immunocytofluorescence studies against S100 and GFAP. The cells were also checked for the lack of contamination with other neural cells by staining against Tuj1, MAP2, Olig2, Nestin and Sox2.

At 24 hours after seeding, astrocytes were infected with GFP or miR-302/367-GFP expressing lentiviral particles and allowed to incubate overnight. Following incubation, at 12 to 16 hours later the astrocyte medium was removed and replaced by fresh astrocyte medium. Forty-eight hours after transfection, astrocytes were transplanted into mice and followed to observe their possible conversion into neurons.

In vitro conversion of astrocytes into neuronal cells was assessed as follows. After removing the medium that contained viral particles at the day post-transfection, neuronal medium that included DMEM-F12 (Invitrogen), 2% N2 (Invitrogen), 0.5% FCS and brain-derived neurotrophic factor (BDNF,20 ng/ml) was added to the cells. The neuronal medium was changed on alternate days.

### Interventions

All animals were anesthetized by intraperitoneal (i.p.) injection of ketamine (70 mg/kg) and xylazine (10 mg/kg) after which they were secured in a stereotaxic apparatus (Steolting, USA). Prior to the surgery, animals received local subcutaneous injections of lidocaine to eliminate any possible pain sensation. Injection needles were stereotaxically inserted into the left striatum (AP: +0.6 mm from the bregma, L: 2.5 mm, DV: 2.5 mm from the dura). We injected 3 μl of medium that contained the viral particles over 5 minutes; for an additional 5 minutes the needle was kept in place to prevent reflux of the injection medium. Following the surgery, animals were locally treated with tetracycline to prevent postoperative infection. Animals were monitored daily for their health condition. We did not observe any incidental death following the interventions. When required, daily VPA (300 mg/kg i.p. as sodium salt) was given to the animals from 4 days prior to viral particle injection up to 7 or 14 days after viral particle administration.

There were six experimental groups with 3 mice per group used for the in vivo reprogramming studies. Two groups of animals received focal injections of control lentiviral particles that expressed GFP as the reporter and i.p. injections of VPA from 4 days prior to viral particle injection until 7 or 14 days post-injection. Groups 3 and 4 received focal injection of viral particles that expressed the miR-302/367+GFP cluster. These groups were evaluated 7 or 14 days post-injection, respectively. Groups 5 and 6 received focal injections of viral particles that expressed the miR-302/367+GFP cluster and VPA injections as mentioned for groups 1 and 2.

For astrocyte transplantation, 300,000 human astrocytes were concentrated in 3 μl of DMEM and transplanted into the mouse striatum two days after transfection. Cyclosporine-A (Novartis) 15 mg/kg was injected i.p. from two days before cell engraftment as an immunosuppressant. Immunosuppression was continued throughout the course of the experiments.

### Immunostaining and histological investigations

For immunohistofluorescence investigations, animals were deeply anesthetized using a combination of ketamine (70 mg/kg) and xylazine (10 mg/kg). Animals were transcardially perfused with 0.1 M phosphate buffered saline (PBS) and 4% paraformaldehyde in PBS, respectively. The animal brains were carefully removed and submerged overnight in the same fresh fixative. The next day, tissues were immersed in 30% sucrose for 24–48 hours. Brains were frozen in a cryostat embedding medium (Bio-Optica, Italy) at -22°C. Coronal sections (8 μm thickness) were prepared by using a cryostat instrument (Histo-Line Laboratories, Italy), then permeabilized with 0.2% Triton X-100, and blocked with 10% normal goat serum for 1 hour. The sections were incubated with primary antibodies overnight at 4°C. After extensive washing with PBS and one hour incubation with appropriate fluorescent labeled secondary antibodies, the preparations were washed again and coverslipped using a mounting medium (Santa Cruz, sc-24941) that contained 4',6-diamidino-2-phenylindole (DAPI) as the nuclear stain. Cells were counted by selecting at least five sections from the injection area for each animal. Cell counts were averaged as the cell number per section for each animal after which we calculated the average for three animals in each experimental group.

Cultured cells were washed three times with PBS, then fixed with pre-cold 4% paraformaldehyde for 10 minutes at room temperature. After extensive washing with PBS for three times, cells were permeabilized with 0.2% Triton X-100 for 20 minutes, then blocked in 10% normal goat serum for 1 hour. The appropriate concentrations of primary antibodies were added and cells were kept at 4°C overnight, then washed for three times with PBS. After the addition of secondary antibodies and incubation for 30 minutes, the cells were again extensively washed and coverslipped as previously mentioned. The slides were investigated under fluorescence microscope (Olympus BX51) and photographed using a DP-72 camera. S1 Table lists information for the primary and secondary antibodies used in this experiment.

We used hematoxylin and eosin staining to assess the possibility of teratoma formation following local injection of the miR-302/367 cluster expressing lentiviral particles. Two months after miR-302/367 injections, the animals were perfused with PBS and paraformaldehyde, respectively and the sections were counterstained with hematoxylin and eosin.

### Whole cell patch clamp recording

Cells were prepared for electrophysiological recording at six weeks after transfection with the miR-302/367 cluster. Glial cells that converted into neurons were seeded on poly D-lysine coated coverslips and transferred to standard artificial cerebrospinal fluid (ACSF) for recording. ACSF was continuously bubbled with 95% O_2_–5% CO_2_ and contained 125 mM NaCl, 3 mM KCl, 1.25 mM NaH_2_PO_4_, 25 mM NaHCO_3_, 10 mM d-Glucose, 2 mM CaCl_2_ and 1.3 mM MgCl_2_. Cells were incubated for 30 minutes at room temperature (23–25°C) before transfer to a submerged recording chamber. The recording chamber was mounted on a fixed-stage upright microscope (Axioskop 2 FS MOT; Carl Zeiss, Göttingen, Germany) and continually perfused at 1.5–2.5 ml/min with standard ACSF at room temperature (23–25°C). Cells were visualized using an IR-CCD camera (IR-1000, MTI, USA) with a 40× water immersion objective lens. Recording microelectrodes (1.5-mm outer diameter, borosilicate glass, GC150-11; Harvard Apparatus, Edenbridge, UK) were pulled using a horizontal puller (P-97, Sutter Instrument, Novato, CA, USA) and filled with an intracellular solution that contained 115 mM K-gluconate, 20 mM KCl, 10 mM HEPES, 2 mM EGTA, 10 mM disodium-phosphocreatine, 2 mM MgATP and 0.3 mM NaGTP. pH was adjusted to 7.25–7.30 and osmolality to 285–290 mOsm. Electrode tip resistance in the bath was typically 4–6 MΩ and series resistance ranged from 12–25 MΩ. Data were low-pass filtered at 3 kHz and acquired at 10 kHz with a Multiclamp 700B amplifier equipped with a Digidata 1440 A/D converter (Molecular Devices, Sunnyvale, CA, USA). The signal was recorded on a PC for offline analysis with Axon pClamp-10 acquisition software. After establishment of a gigaseal (>2 GΩ), the whole-cell configuration was attained by the application of a brief suction.

### Statistical analysis

The results were expressed as means ± SEM. Mean differences were analyzed using one-way analysis of variance (ANOVA) and Tukey post-hoc. p<0.05 was considered statistically significant.

## Results

### In vivo transduction and characterization of transduced cells

**Fig 1:**
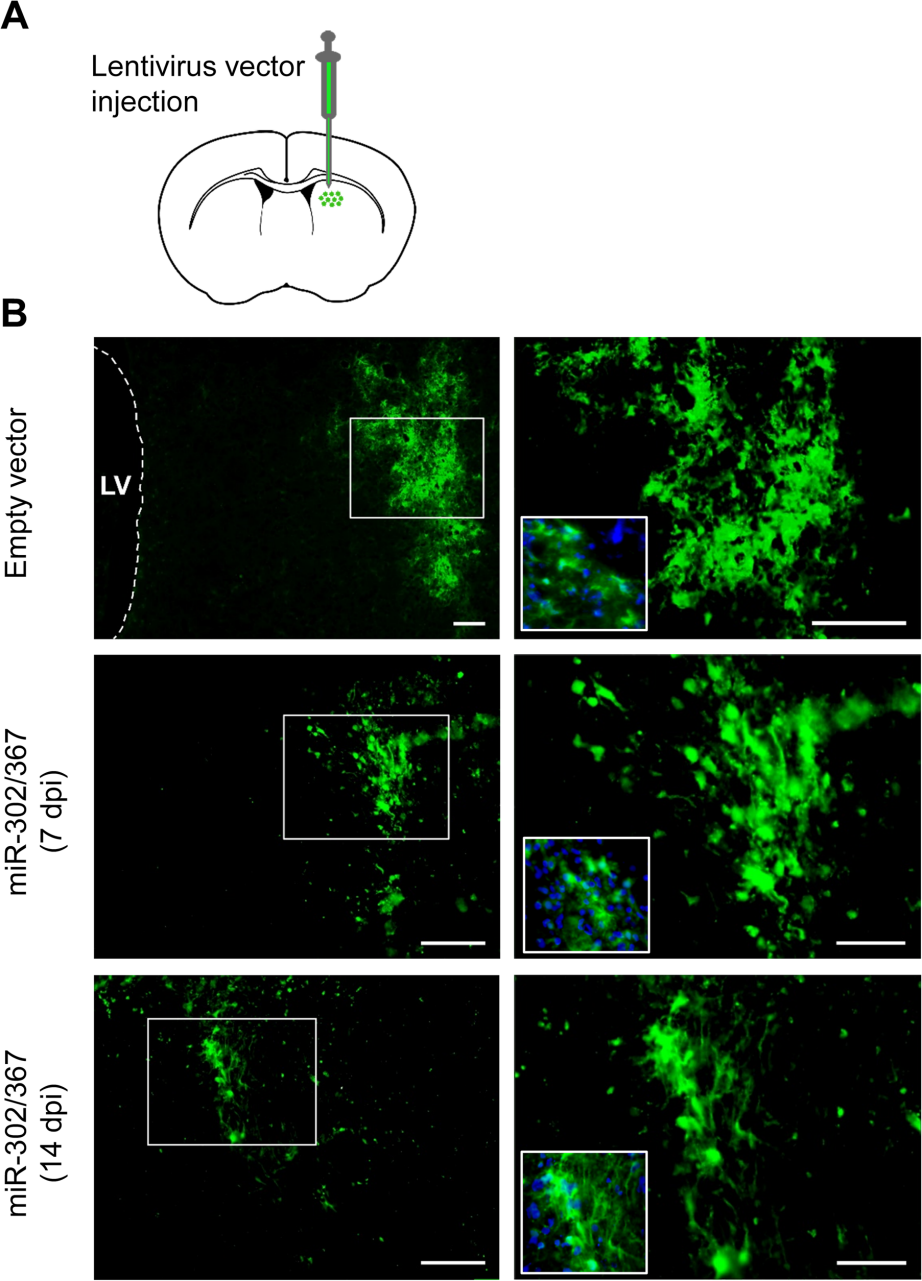
Administration of GFP and miR-302/367 expressing lentiviral particles into the striatum and the distribution of transduced cells. Transduction and continuous expression of vectors were confirmed by injecting a GFP expressing empty lentiviral vector and lentiviral particle that included the miR-302/367+GFP cluster. Scale bar: 100 μm.

miR-302/367 expressing lentiviral particles were injected into the mice striata to induce transdifferentiation of targeted cells into neuroblasts. We chose the lateral striatum because of its distant location from the lateral ventricle germinative zone in order to eliminate the possibility of detecting endogenous neural progenitor cells instead of induced neuroblasts. In order to investigate the type of the cells transfected by the lentiviral particles, GFP expressing lentiviral particles as the control was packed and injected into the striatum. The same pattern of GFP expression was detected when miR-302/367-GFP expressing lentiviral vectors were used. Fig 1 shows the expression of GFP at 7 and 14 days post-injection (dpi) and confirms constitutive expression of the injected vector during the study.

**Fig 2:**
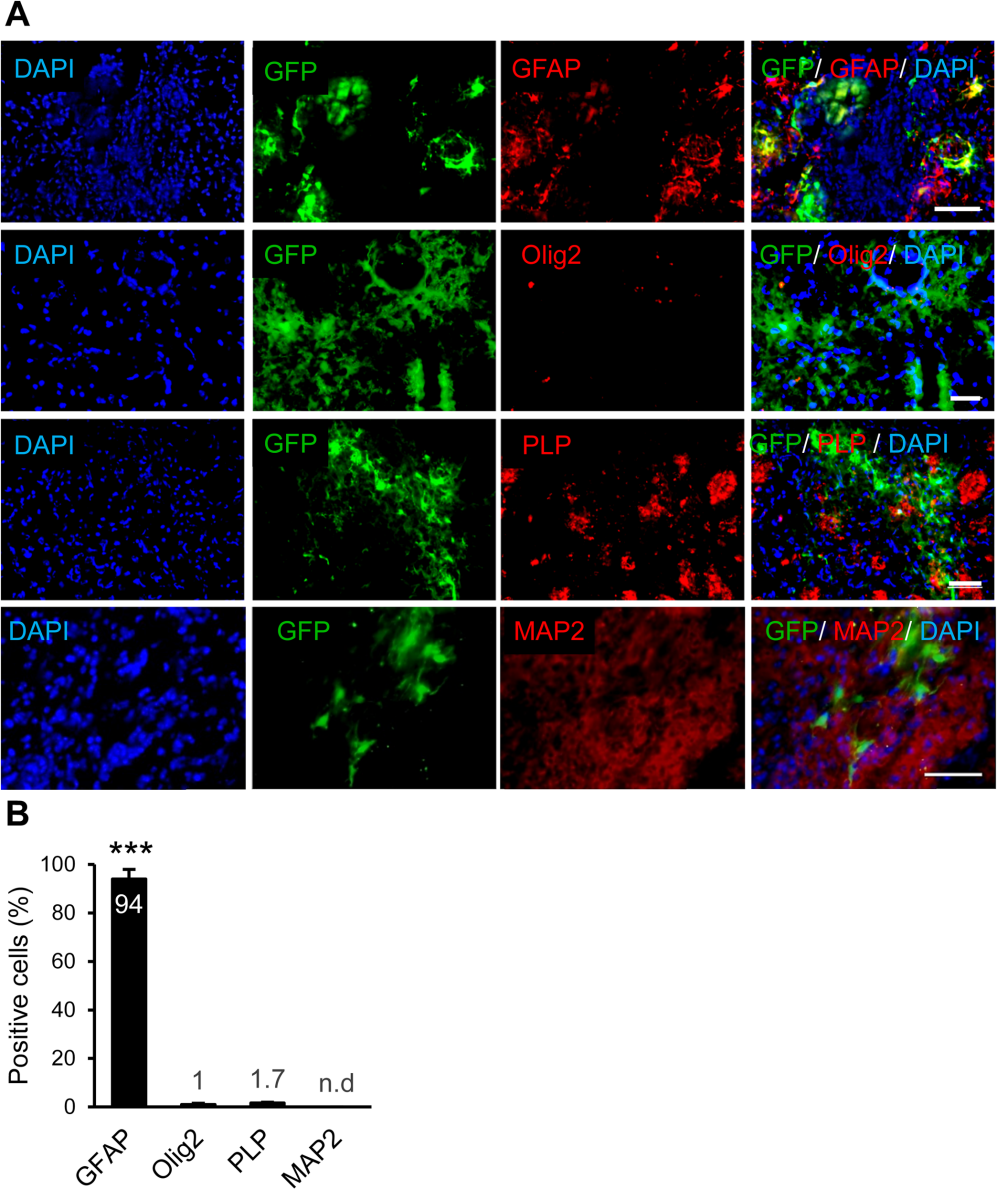
Determining of the fate of cells transduced with GFP expressing vector. (A) Immunofluorescence studies against astrocytes, oligodendrocytes, neurons and mature myelinating cell markers showed that GFAP^+^ astrocytes were the main population of transduced cells. (B) Quantification result of immunostaining against different markers**.** ***p<0.001, n = 3 mice per group. Scale bar: 50 μm.

The brain samples were sectioned and evaluated at 10 dpi. The sections were stained for GFAP, Olig2, PLP and MAP2 to assess their co-expressions with GFP (Fig 2). The results showed that astrocytes around the stab injury were reactivated and GFAP^+^. The majority of GFP^+^ cells expressed GFAP which indicated their astrocyte fate. A few number of GFP^+^ cells expressed Olig2, an oligodendrocyte progenitor marker. We could not detect any notable co-expressions of GFP and PLP as markers of mature myelinating cells or co-expressions of GFP and MAP2 as neuronal markers (Fig 2A). Fig 2B shows the quantitative data for the percentage of GFP expressing cells which expressed GFAP (94.0%), Olig2 (1.0%), PLP (1.7%) or MAP2 (none detected). Therefore, astrocytes were the main population of cells which received the lentiviral particles. The possible transfection of endogenous neural stem cells and neuroblasts was rule out by evaluating the expression of Nestin and DCX in transduced (GFP+) cells (S1 Fig).

### Neuronal fate of in vivo transduced cells

**Fig 3:**
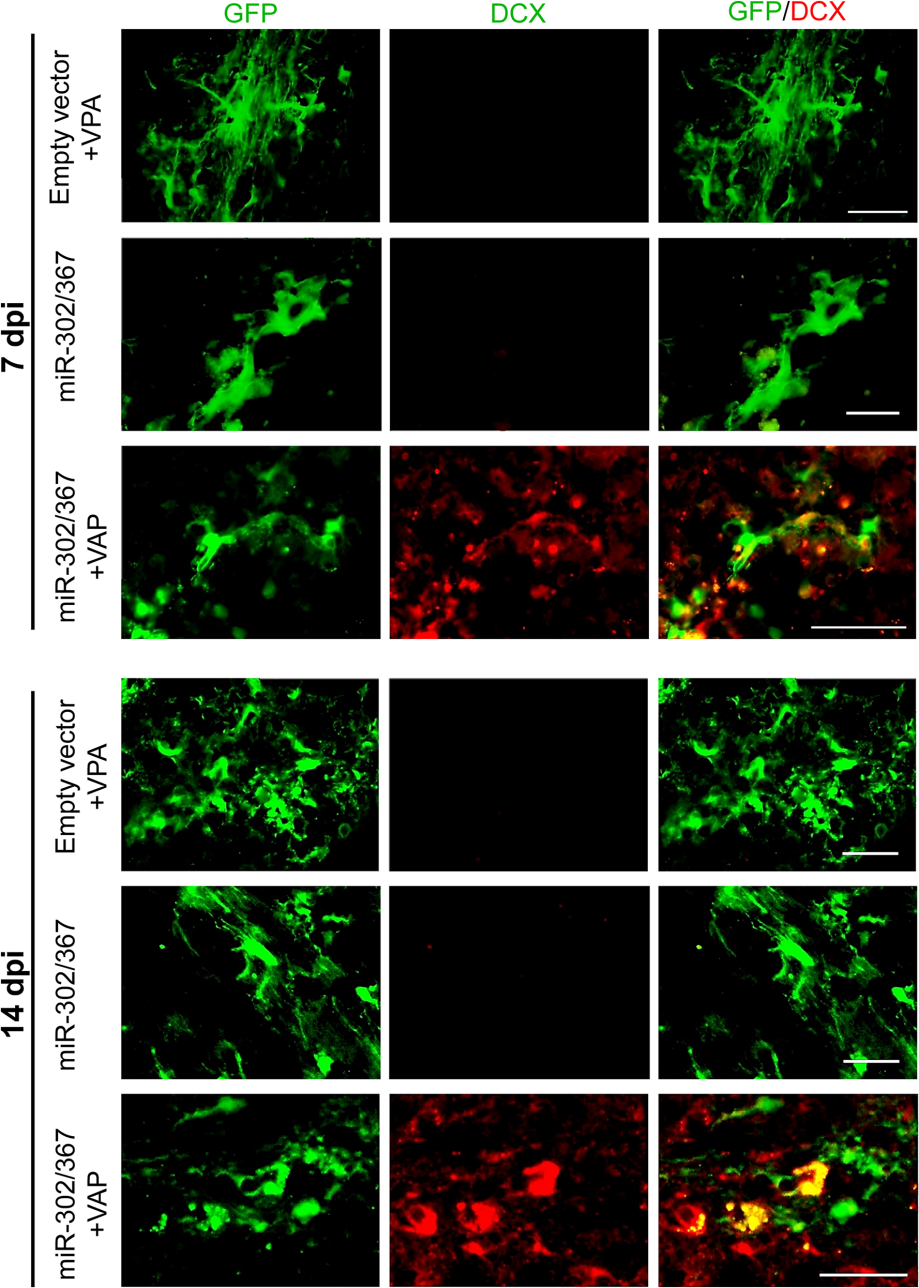
miR-302/367 and valproic acid (VPA) converted the transducted cells into neuroblasts. Doublecortin (DCX^+^) cells were detected at 7 and 14 days post-injection (dpi) in animals pre-treated with VPA that afterwards received miR-302/367. n = 3 mice per group. Scale bar: 30 μm.

Following focal administration of the miR-302/367 cluster into the striatum, we studied the fate of transfected cells by specific staining against doublecortin (DCX) as a neuroblast marker. The administration of empty vector (GFP expressing vector) and/or miR-302/367 cluster vector did not cause the conversion of transfected (green) cells into neuroblasts during 2 weeks (Fig 3). However, the combination of miR-302/367 and VPA resulted co-expression of DCX^+^/GFP^+^ cells at both 7 and 14 dpi (Fig 3) which implied conversion of astrocytes into neuroblasts.

**Fig 4:**
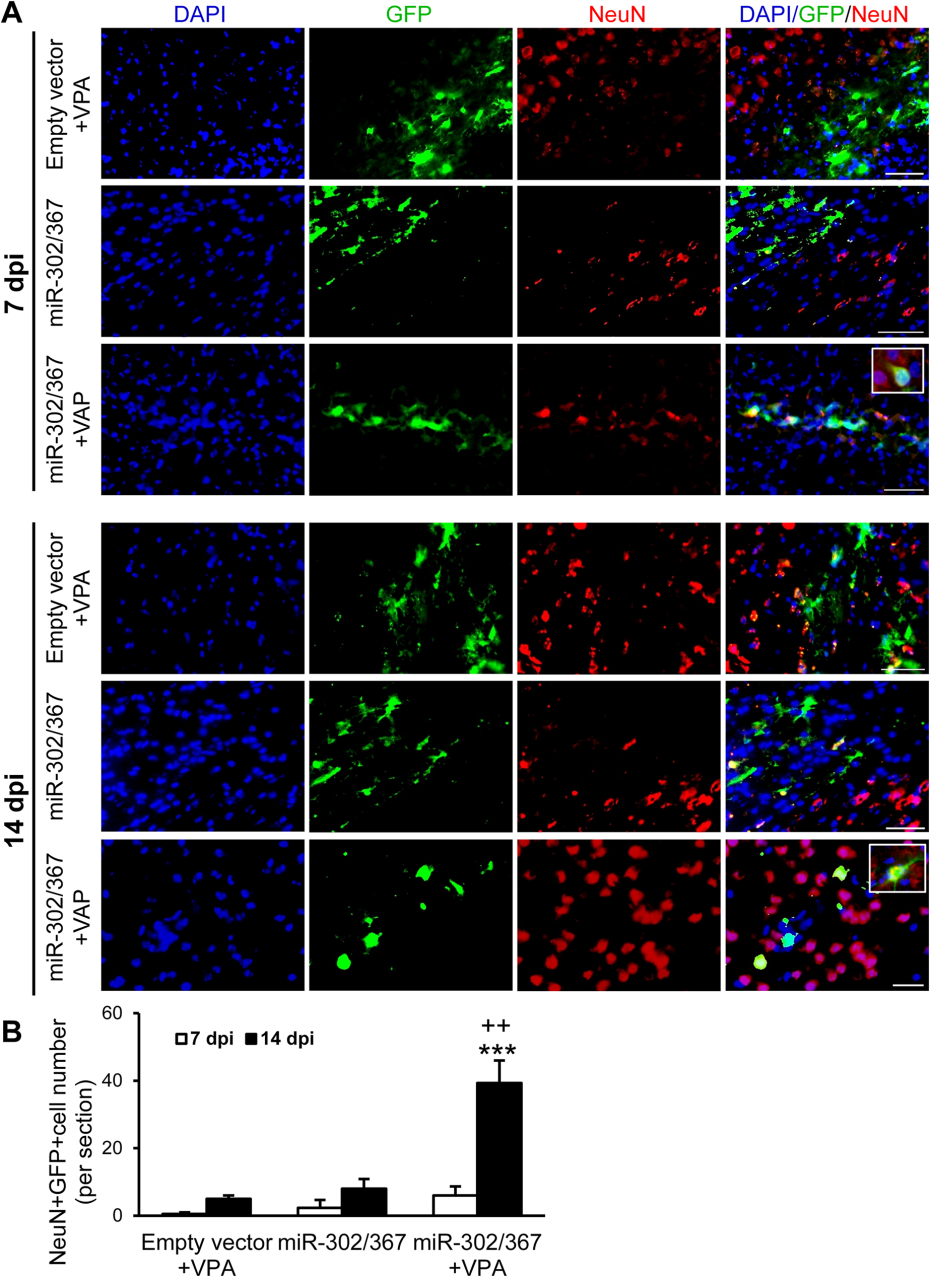
A number of cells transduced with the miR-302/367+GFP cluster following valproic acid (VPA) pre-treatment showed neuronal fate as determined by immunostaining against NeuN. **(A)** Animals received miR-302/367+GFP expressing vectors. VPA showed GFP^+^ cells that expressed NeuN. **(B)** Quantification of immunostaining data showed that GFP^+^/NeuN^+^ cells were produced in the miR-302/367+VPA group, particularly at 14 days post-injection (dpi). ***p<0.001 compared to 7 dpi and ^++^p<0.01 compared to miR-302/367 and VPA groups at 14 dpi. n = 3 mice per group. Scale bar: 50 μm.

Then, we assessed neuronal differentiation of transfected cells by checking for co-localization of GFP and NeuN, a mature neural cell marker. A few GFP^+^/NeuN^+^ cells were detected within the brains of mice that received control vector+VPA (average cell count: day 7, 0.5; day 14, 8) or miR-302/367 expressing vector (average cell count: day7, 5; day 14, 6) (Fig 4A). Animals which received both VPA and miR-302/367 expressing vector had higher numbers of GFP^+^/NeuN^+^ cells especially at day 14 (average cell number: 39.33) (Fig 4). The number of GFP^+^ cells which expressed NeuN significantly increased in animals treated with the miR-302/367 cluster and VPA, especially at day 14. (p<0.001 vs. same group at day 7, p< 0.01 vs. both miR302/367 and empty vector+VPA groups at day 14). These data showed the neuronal differentiation of cells induced by miR302/367+VPA.

### Astrocyte conversion to neurons without reprogramming to the pluripotent stage

**Fig 5:**
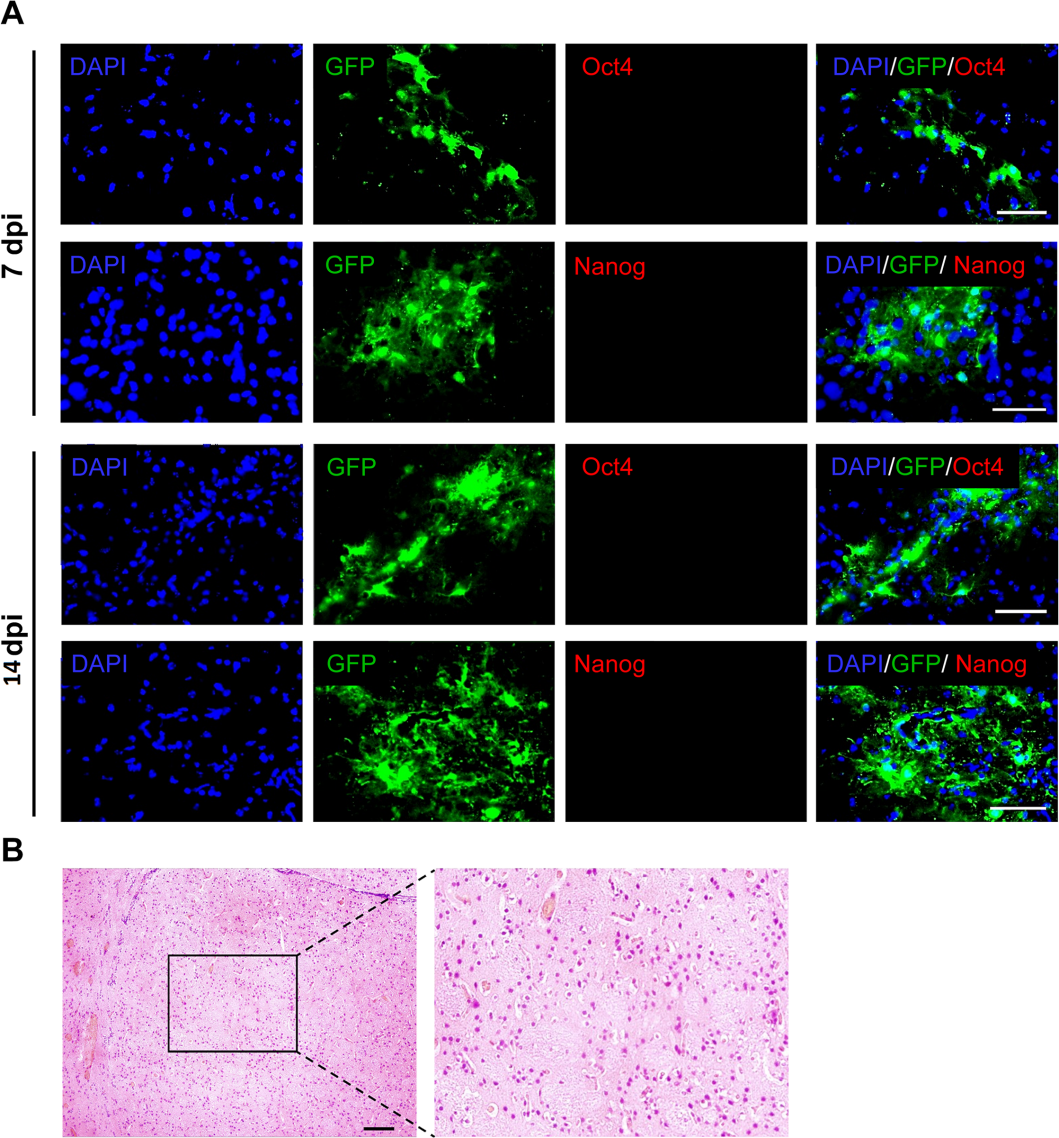
Conversion of astrocytes into neuronal cells which did not pass through the pluripotent stage. **(A)** Expressions of Oct4 and Nanog genes as pluripotent markers were not detected following miR-302/367+ valproic acid (VPA) treatment. n = 3. Scale bar: 50 μm. (B) The brain tissue of animals treated with the miR-302/367 cluster were investigated for the teratoma formation. No teratoma formation was observed at 60 days post-injection (dpi). n = 2. Scale bar: 50 μm.

We sought to determine whether induced neuroblasts resulted from direct reprogramming or pluripotent reprogramming followed by neural differentiation by investigating the expressions of pluripotency markers such as Oct4 and Nanog at both 7 and 14 dpi. According to Fig 5A, we could not detected any Oct4 and Nanog positive cells in the sections obtained from mice treated with both the miR-302/367 cluster and VPA. Two months after injection of viral particles that expressed miR-302/367 and VPA, the striata areas were investigated for teratoma formation. We observed no teratoma formation within the injected areas of the brains (Fig 5B).

### In vivo conversion of engrafted human astrocytes to neuroblasts by miR-302/367

**Fig 6:**
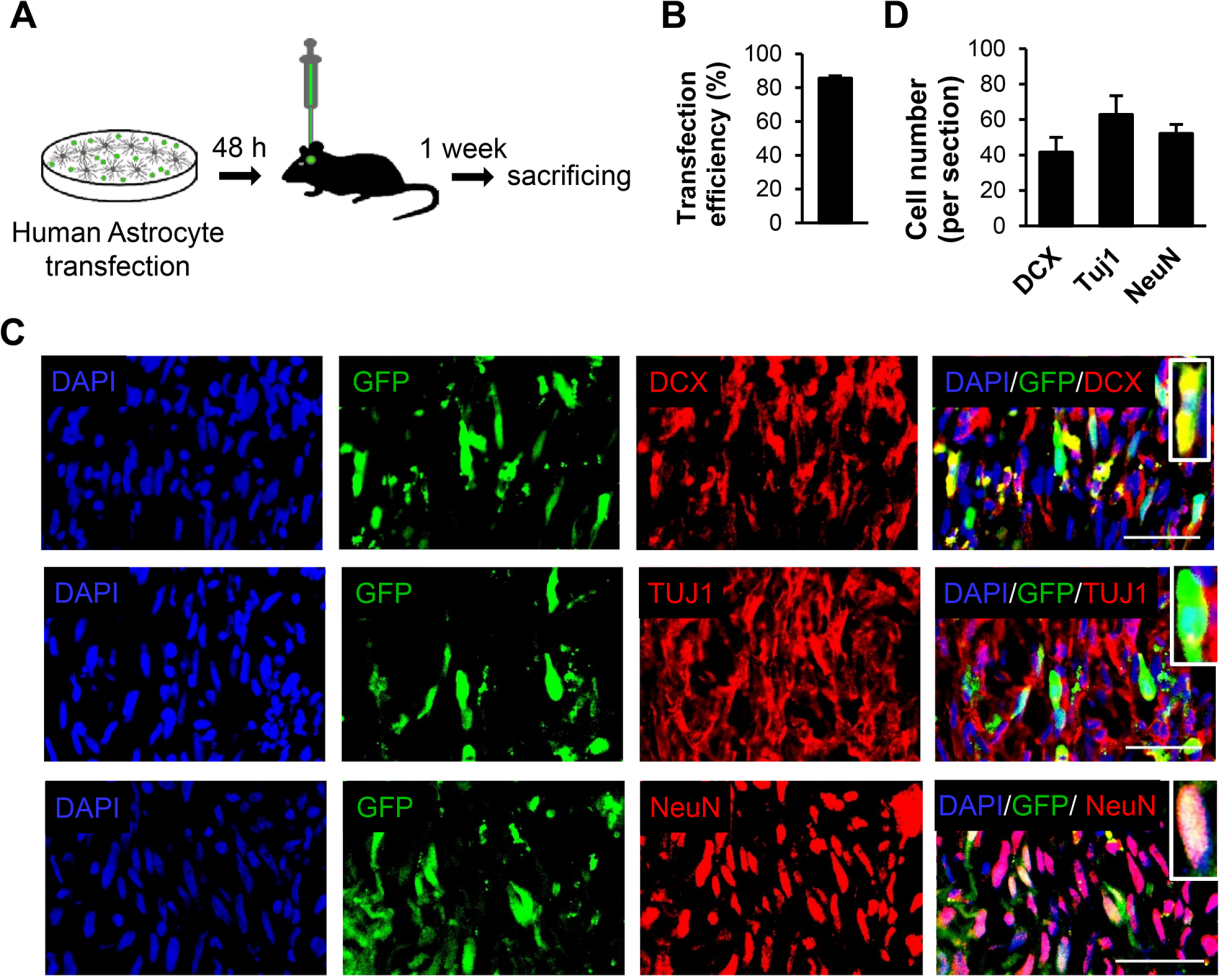
Human astrocytes were transduced with the miR-302/367+GFP expressing vector in vitro and then transplanted into mice striata. **(A)** Schematic diagram of the experiment. **(B)** Transfection efficiency. **(C)** A number of transplanted human astrocytes expressed neuroblasts and neuronal markers 9 days after transduction (7 days after transplantation). **(D)** Quantification of cells immunostained with anti-doublecortin (DCX), anti-TUJ1 and anti-NeuN antibodies. n = 3. Scale bar: 50 μm.

In order to prove in vivo conversion of astrocytes to neuronal cells, we transfected human cultured astrocytes with miR-302/367-GFP expressing viral particles which were subsequently transplanted into the striatum (Fig 6A). More than 80% of astrocytes became transduced with miR-302/367 and expressed GFP (Fig 6B). Nine days after transduction of astrocytes with miR-302/376, immunofluorescence staining was performed to determine the fate of the engrafted cells. We detected a number of GFP^+^ cells that expressed DCX, TUJ1 and NeuN in the mice striata (Fig 6C). Fig 6D shows the numbers of DCX, TUJ1 and NeuN positive cells within the injected area. This data showed the conversion of induced human astrocytes to neuronal cells by miR302/367 alone.

### In vitro conversion of astrocytes to neuron-like cells by miR-302/367

**Fig 7:**
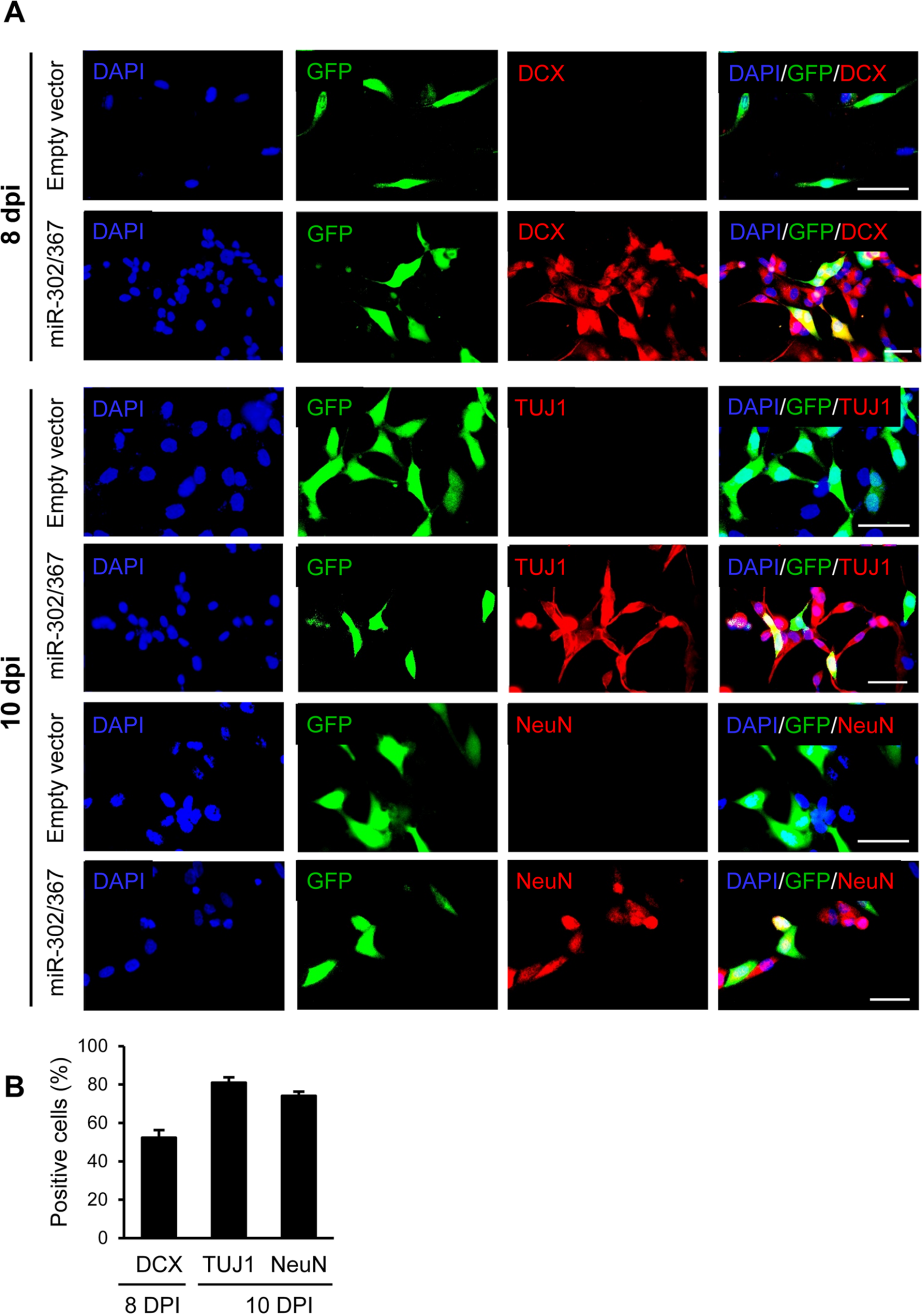
Human astrocytes were converted into neurons by the miR-302/367 cluster in vitro, without pre-treatment with valproic acid (VPA). **(A)** Human induced neurons expressed neuroblast marker [doublecortin (DCX)] and neuronal markers (TUJ1 and NeuN) at 8 and 10 days post in vitro (DPI) when they received the miR-302/367 cluster. **(B)** Quantification of immunostaining data provided in A. Scale bar: 50 μm.

In vitro reprogramming of human astrocytes into neuroblasts was determined by transfection of astrocytes with viral particles that expressed miR-302/367. These cells were followed post-in vitro trans-differentiation. Eight to ten days after transduction, cells were assessed for neuronal marker expressions by immunofluorescence staining. We observed DCX, TUJ1 and NeuN positive cells in the miR-302/367+GFP treated cultures which implied conversion of astrocytes into neuroblasts and neuronal cells. The cell cultures that received GFP-only expressing viral vectors (empty vector) did not contain neuronal markers (Fig 7A). Fig 7B lists the numbers of GFP^+^/DCX^+^, GFP^+^/TUJ1^+^ and GFP^+^/NeuN^+^ cells.

**Fig 8:**
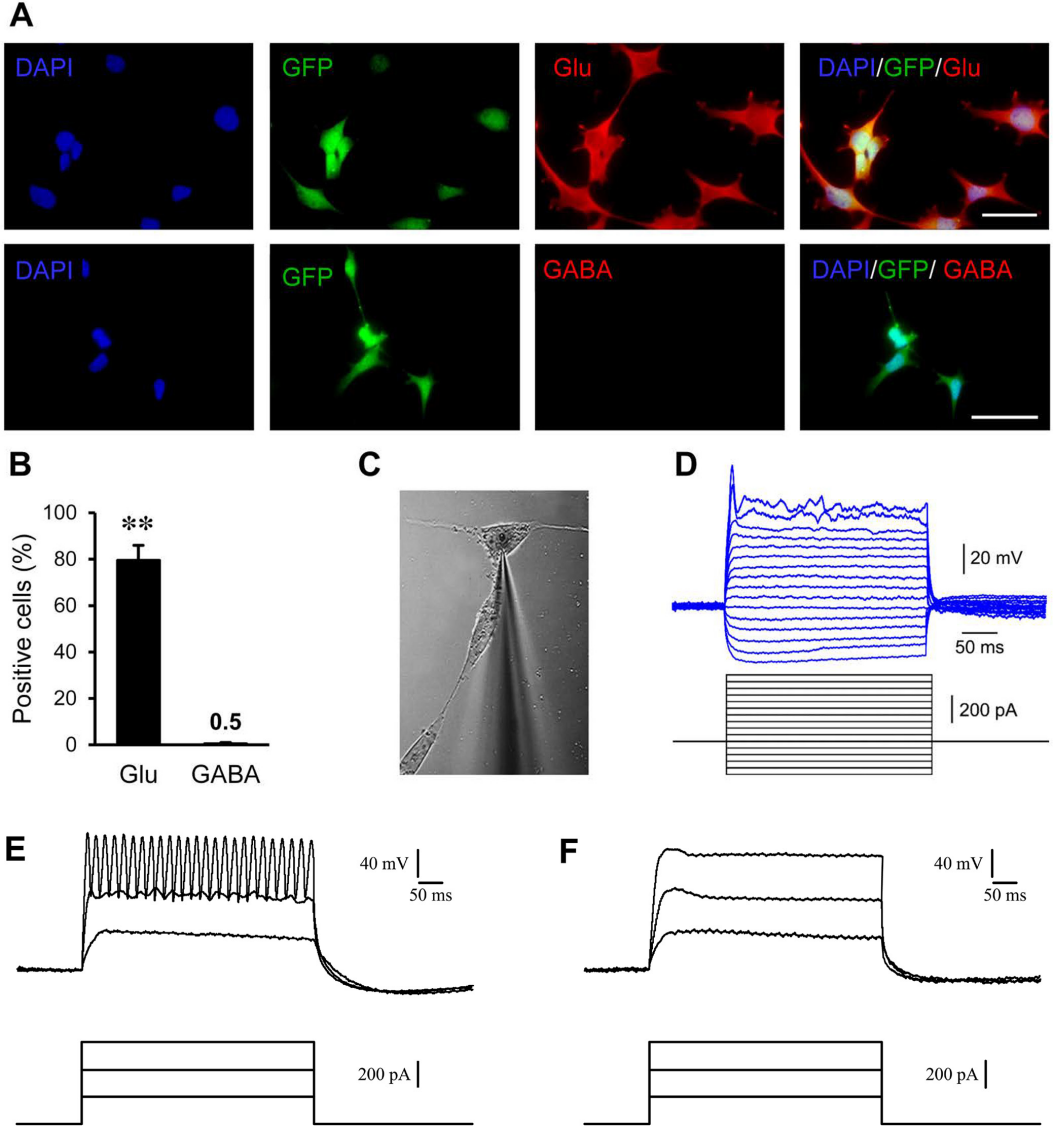
Induced neurons showed mature neuronal properties at six and ten weeks after transduction in vitro. **(A)** The majority of neurons were positive for glutamate (Glu) as a marker for excitatory neurons at six weeks post-transduction. **(B)** Quantification of immunostaining data provided in A. **(C and D)** Whole cell patch clamp recording from induced neuron-like cells at six weeks post-transduction (n = 25) showed single action potential-like spikes. **(E)** Similar recording at ten weeks post-transduction (n = 10) showed repetitive spike firing. **(F)** Treatment with 1 μM TTX as sodium channel blocker, inhibited spike firing. **p< 0.01. Scale bar: 50 μm

At six weeks after transfection of astrocytes with the miR-302/367 cluster, we checked for the expressions of markers of differentiated neurons. Cells were cultured on coverslips and stained against glutamate (Glu) and GABA. As Fig 8A shows, approximately 80% of cells expressed glutamate which implied that glutamergic differentiation was the main phenotype. Quantification of data showed a high percentage of Glu^+^ and a very low percentage of GABA^+^ cells (Fig 8B).

Functional evaluation of induced neurons was assessed by patch clamp recording at six and ten weeks after transfection. At six weeks post-transfection, we used 25 neuron-like cells to evaluate resting membrane and action potentials. The current clamp mode was used to check for changes in membrane voltage following injection of depolarizing and hyperpolarizing currents. Recorded resting membrane varied between -20 to -30 mV. Before injection of currents, voltage was adjusted to -65 mV. We observed a single spike following current injection in the induced neurons but not repetitive spike firing. The maximum amplitude of spikes was around 40 mV (Fig 8C and 8D). This data showed that 6 weeks after in vitro induction of human astrocytes, the produced neurons mainly expressed the phenotype of excitatory neurons and showed preliminary electrophysiological properties of neurons. At ten weeks post-transfection, we evaluated 10 neuron-like cells for their electrophysiological characteristics. These cells showed a resting membrane potential between -30 to—40 mV and when injected with the mentioned depolarizing current, showed repetitive spike firing (Fig 8E). Pretreatment with TTX (1μM) as a sodium channel blocker disappeared the firing of repetitive spikes (Fig 8F) which imply for the presence of sodium current in neuron-like cells at 10 weeks post-transfection.

## Discussion

We showed that application of the miR-302/367 resulted in reprograming of adult mouse human astrocytes into neuroblasts both in vivo and in vitro. Recently it has been shown that astrocytes can be reprogrammed into functional neurons by a single transcription factor, Sox2, in vivo [17]. Following in vivo injection of Sox2, they reported the presence of induced neuroblasts at four weeks after intra-striatal injections of lentiviral particles. Guo et al. have shown that astrocytes could be converted efficiently to neurons by a specific transcription factor, NeuroD1. These works revealed that human astrocytes could be trans-differentiated into neurons in vivo.

Based on our knowledge the current study is the first report that shows which a microRNA can convert astrocytes into neuroblasts. The miR-302/367 cluster is involved in early embryonic development and expressed in NSCs. The pluripotency genes, Oct4, Sox2 and Nanog increase the expression of miR-302/367 via binding to its promoter. Interestingly, in a positive feedback loop miRNA-302/367 enhances the expression of the above mentioned reprogramming factors. Considering the previous reports on the effectiveness of Oct4, Sox2 and Nanog in inducing astrocytes into neurons, this positive loop may explain a possible mechanism for miRNA-302/367 induced neuronal fate from astrocytes [27, 28, 35]. The effect of microRNAs can be reproduced by miR-mimic sequences which do not integrate into the genome and therefore promise the possibility of designing a safe approach for in vivo induction of neural progenitors. Previously miR-124 and miR-9 have been used for in vitro induction of neurons from fibroblasts [6].

In a recent study by Heinrich et al., it was demonstrated that conversion of NG2^+^ glial cells into neurons inside the mouse cortex was impossible in the absence of a stab wound. In other words reactivated cells seemed more susceptible to fate conversion which might promise selective conversion of cells within the glial scar rather than cells with normal physiological activity. Up-regulation of a number of neurogenic factors that release after a stab wound might facilitate reprogramming [36]. After a stab injury, different types of glial cells become and proliferate. Among these cells, astrocytes are the major cell type that receives the lentiviral particles [16, 17]. Our data have also shown that astrocytes were the main type of cells that received viral particles. Following induction, the highest number of DCX^+^ cells was observed one week post-induction while the number of NeuN^+^ cells were visualized two weeks post-induction. This finding might imply conversion of DCX^+^ neuroblasts into NeuN^+^ neurons.

In the current study, induced neuroblasts were observed earlier compared to previous reports that used Sox2 [17]. Another remarkable point in our study was the pivotal role of VPA. miR-302-367 alone could not efficiently reprogram mouse astrocytes. Previous reports suggested that VPA as a histone deacetylase inhibitor had an important role in the chromatin remodeling process and increased the efficiency of reprogramming in mouse fibroblasts [25]. miR-302/376 induced neurons might be the result of pluripotent reprogramming of glial cells towards iPSCs and their subsequent differentiation to neuronal cells, or the result of direct conversion of astrocytes into neuroblasts. Following in vivo application of miR-302 we checked for the expression of pluripotency markers Oct4 and Nanog and did not detect positive cells. These induced neurons appeared to be reprogrammed directly from astrocytes without passing through the pluripotent stage.

Although the astrocytes were the major transfected cells after in vivo injection of viral particles, other cell types might also receive miR-302/367 and undergo reprogramming. Although our data could not rule out this possibility, we attempted to prove conversion of these astrocytes. We transfected human astrocytes in vitro and then transplanted them into mice striata. We observed a remarkable population of GFP^+^ transplanted cells that expressed neuroblast and neural cells markers around the injection site.

In addition, we showed that human astrocytes could reprogram into neurons by the miR-302/367 cluster alone in neuronal differentiation medium. The presence of VPA for transdifferentiation of these cells was not required which was suggested to be the result of lower histone deacetylase activity in human cells [25]. Following in vitro neuronal induction of astrocytes by using the miR-302/367 cluster, some neuroblasts and neurons were detected that did not express GFP. The GFP signal in these cells might be quenched by chromatin remodeling during fate conversion or their physiological status.

Six weeks after in vitro induction and culturing of astrocytes, partially mature induced neurons were evaluated for their fate. The induced neurons were mostly excitatory to anti-Glu. Electrophysiology assessments using patch clamp recording demonstrated a resting membrane and the ability to fire singe spikes. Ten weeks after in vitro transduction, neuron-like cells showed sodium channel mediated repetitive spike firing.

Along with recent reports on in vivo reprogramming of astrocytes to neuroblasts and neurons, this study introduced a new approach for the production of neuroblasts from astrocytes within glial scars as a chronic outcome of neural injuries. The use of viral particles for reprogramming and some integration in host genome limited therapeutic application of these methods. The effect of miRNAs could be reproduced by miR-mimic sequences which appeared to be safer method for therapeutic applications.

## Supporting Information

S1 Fig Lack of Nestin and DCX expression in cells transduced with GFP expressing vector. (PDF)

S1 Table List of primary and secondary antibodies used in this study. (DOCX)
