# Supplementary material for: MicroRNA-Mediated In Vitro and In Vivo Direct Conversion of Astrocytes to Neuroblasts
Source: PLoS One. 2015 Jun 1;10(6):e0127878. doi: 10.1371/journal.pone.0127878 (PMC4451260; doi:10.1371/journal.pone.0127878)
Supplement: S1 Fig — (PDF) [file pone.0127878.s001.pdf]

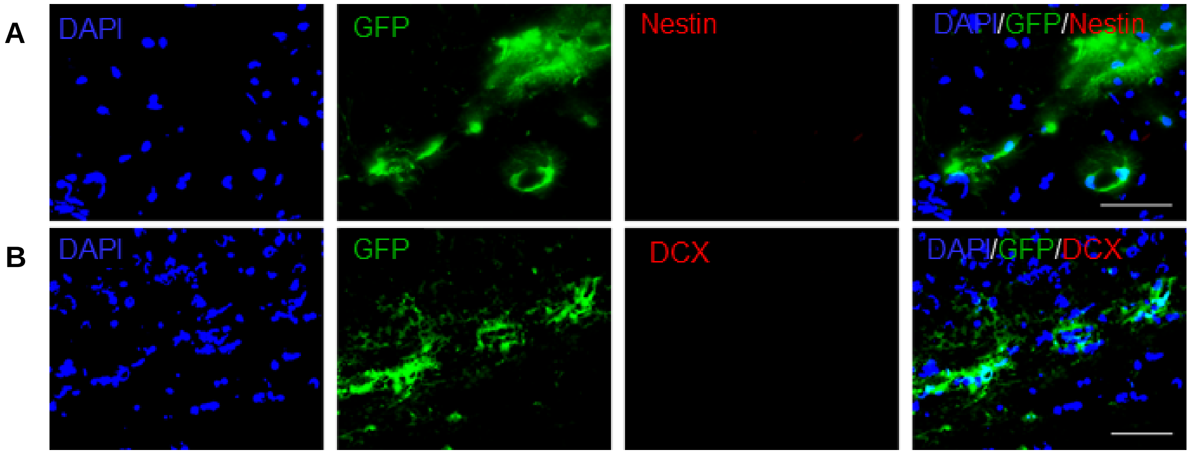

S1 Fig. Lack of Nestin and DCX expression in cells transduced with GFP expressing vector. Immunofluorescence studies against neural stem cell (A) and neuroblast (B) markers showed that transfected cells did not express Nestin and DCX. This finding shows that GFP+ neuronal cells appeared following in vivo transduction with miR-302/367, were not originated from endogenous neural stem cells and neuroblasts. Scale bar: 50 µm.
