# Supplementary material for: MicroRNA-Mediated In Vitro and In Vivo Direct Conversion of Astrocytes to Neuroblasts
Source: PLoS One. 2015 Jun 1;10(6):e0127878. doi: 10.1371/journal.pone.0127878 (PMC4451260; doi:10.1371/journal.pone.0127878)
Supplement: S1 Table — (DOCX) [file pone.0127878.s002.docx]

**S1 Table: List of primary and secondary antibodies used in this study.**

| ***Target molecule*** | ***Species isotype*** | ***Label*** | ***Company*** | ***Final concentration*** |
| --- | --- | --- | --- | --- |
| GFAP | Rabbit polyclonal IgG | - | Dako, Z0334 | 1:300 |
| Olig2 | Rabbit polyclonal IgG | - | Abcam, Inc. ab9610 | 1:200 |
| PLP | Rabbit polyclonal IgG | - | Abcam, Inc. ab28468 | 1:100 |
| MAP2 | Mouse monoclonal IgG | - | Sigma-Aldrich, M1406 | 1:200 |
| DCX | Rabbit polyclonal IgG | - | Abcam, Inc. ab18723 | 1:500 |
| NeuN | Rabbit monoclonal IgG | - | Abcam, Inc. ab177487 | 1:500 |
| Tubulin III | Rabbit polyclonal IgG | - | Abcam, Inc. ab6046 | 1:200 |
| Oct4 | Mouse monoclonal IgG | - | Santa Cruz, Sc-5279 | 1:25 |
| Nanog | Rabbit polyclonal IgG | - | Abcam, Inc. ab80892 | 1:50 |
| Glu | Rabbit polyclonal IgG | - | Sigma-Aldrich, G6642 | 1:250 |
| GABA | Rabbit polyclonal IgG | - | Millipore, AB131 | 1:250 |
| Nestin | Rabbit polyclonal IgG | - | Abcam, Inc. ab27952 | 1:200 |
| Rabbit IgG | Goat anti-rabbit | Alexa Fluor® 594 | Abcam, Inc. ab150080 | 1:1000 |
| Mouse IgG | Goat anti-mouse | Alexa Fluor® 568 | Life Technologies, A11004 | 1:1000 |
